# Effect of Nonsteroidal Anti‐Inflammatory Drugs on Sacroiliac Joint Inflammation, as Seen on Magnetic Resonance Imaging, in Axial Spondyloarthritis

**DOI:** 10.1002/acr.25581

**Published:** 2025-09-10

**Authors:** Gareth T. Jones, Alexander N. Bennett, Raj Sengupta, Pedro M. Machado, Helena Marzo‐Ortega, Lorna Aucott, Margaret A. Hall‐Craggs, Timothy J. P. Bray, Alan Bainbridge, Ruaridh M. Gollifer, Gary J. Macfarlane

**Affiliations:** ^1^ University of Aberdeen Aberdeen United Kingdom; ^2^ Defence Medical Rehabilitation Centre, Stanford Hall, United Kingdom, and Imperial College London London United Kingdom; ^3^ Royal National Hospital for Rheumatic Diseases and University of Bath Bath United Kingdom; ^4^ University College London, University College London Hospitals NHS Foundation Trust, and London North West University Healthcare NHS Trust London United Kingdom; ^5^ Leeds Teaching Hospitals NHS Trust and University of Leeds Leeds United Kingdom; ^6^ University College London and University College London Hospitals NHS Foundation Trust London United Kingdom; ^7^ University College London Hospitals NHS Foundation Trust London United Kingdom

## Abstract

**Objective:**

Imaging evidence of active sacroiliitis is important for diagnosis, classification, and monitoring of axial spondyloarthritis (axSpA). However, there is no consistent guidance on whether patients should temporarily stop nonsteroidal anti‐inflammatory drugs (NSAIDs) before magnetic resonance imaging (MRI). The aim of this study was to determine whether NSAIDs lead to an underestimation of active sacroiliitis, as observed using MRI.

**Methods:**

Adults with axSpA were recruited from rheumatology clinics and undertook NSAID washout for one to two weeks before a sacroiliac joint MRI scan. Images were read by two independent readers and adjudicated by a third if required. Those who had a positive result for active sacroiliitis, as per internationally recognized criteria, underwent a second scan six weeks after recommencing daily NSAIDs. We determined the proportion of participants who had a negative scanning result while taking NSAIDs after a previous positive result when NSAID‐free. Images were also scored using semiquantitative methods comprising lesion size and intensity, and a subset of participants underwent quantitative MRI (qMRI) to provide an objective evaluation of any inflammatory changes.

**Results:**

From 34 centers across the United Kingdom, 311 participants (median age 42 years; 62% male) were recruited; 286 (92%) completed the NSAID washout and underwent the first MRI scan. From 146 participants with active sacroiliitis, follow‐up scans (while taking NSAIDs) were obtained from 124 (85%), at which point 25 participants had a negative result (20.2%; 95% confidence interval 13.5%–28.3%). Semiquantitative and qMRI methods supported these findings.

**Conclusion:**

One‐fifth of patients showed full resolution of active sacroiliitis lesions when NSAIDs were present. In clinical practice, if patients with axSpA are willing to attempt a one‐ to two‐week NSAID washout before MRI, this should be considered.

## INTRODUCTION

Axial spondyloarthritis (axSpA) is a chronic inflammatory disease predominantly affecting the spine and sacroiliac joints. Two disease phenotypes, radiographic axSpA (r‐axSpA) and non‐radiographic axSpA, are identified based on the presence or absence of structural changes of sacroiliitis on plain radiography. Disease classification has evolved over recent decades, and the now widely accepted Assessment of SpondyloArthritis international Society (ASAS) classification criteria allow for magnetic resonance imaging (MRI) and various clinical features to phenotype patients further.^1^

**SIGNIFICANCE & INNOVATIONS**

- In axial spondyloarthritis, there are no internationally recognized guidelines for whether patients should discontinue anti‐inflammatory medication before sacroiliac joint magnetic resonance imaging (MRI). Practice differs, and evidence is lacking.
- This study demonstrates that a sizable proportion of patients exhibit significant improvements in sacroiliitis in the presence of nonsteroidal anti‐inflammatory drugs (NSAIDs). Improvements in lesion severity were observed in >40% of patients, with one in five showing full resolution.
- Despite concerns about increases in pain during an NSAID washout period, this study also provides evidence that a brief NSAID washout can be tolerated by almost all patients who attempt it.
- Future imaging guidelines should incorporate this evidence and recommend that if a patient is willing to attempt NSAID washout before MRI, they should be encouraged and supported to do this, including provision of other non–anti‐inflammatory medication if required.

MRI is a standard imaging modality in axSpA, and a key factor aiding diagnosis and classification is the presence of bone marrow edema (BME) lesions suggestive of active sacroiliitis. Indeed, in the United Kingdom, the National Institute for Health and Care Excellence guidance for the diagnosis and management of axSpA recommends that diagnosis can be supported using MRI,^2^ and acquisition and interpretation of MRI findings is facilitated by a joint consensus from radiologists and rheumatologists under the auspices of the British Society for Spondyloarthritis.^3^

Nonsteroidal anti‐inflammatory drugs (NSAIDs) are a common first‐line therapy in axSpA, with continuous treatment recommended for persistent symptoms.^4^ Because NSAIDs are readily available without prescription, many patients are, or have been, exposed to these drugs when they first present to rheumatology. A common clinical consideration during axSpA diagnostic workup is whether NSAIDs should be withdrawn at the time of imaging; it is plausible that their presence could limit the ability to observe inflammation. Currently, no guidelines exist for whether NSAIDs should be discontinued before MRI, and evidence is still lacking. A small UK study examined patients who met the modified New York criteria for ankylosing spondylitis^5^ and who were eligible for tumor necrosis factor (TNF) inhibition.^6^ Among nine patients with follow‐up MRI, 8 of 22 inflammatory sacroiliac joints lesions had resolved following six weeks of NSAIDs (etoricoxib), although the study was too small to be confident about any conclusions. Another small study of 20 Belgian patients with newly diagnosed axSpA found that none had a normal sacroiliac joint MRI finding after six weeks of optimal‐dose NSAIDs, although the intensity of lesions was decreased.^7^ Yet real‐world evidence shows that NSAIDs can lead to near resolution of severe BME lesions in presumed reactive sacroiliitis over an eight‐week period.^8^

We hypothesized that among patients with axSpA, the use of NSAIDs would mask underlying active sacroiliitis when observed using MRI. Not only is this important in terms of diagnosis, but it also has potential implications for treatment (correct diagnosis is required to ensure appropriate management) and research (to determine eligibility for some clinical trials).

The primary objective of the current study was to determine the proportion and characteristics of patients with axSpA with active sacroiliitis on MRI in the absence of NSAIDs, which resolved after NSAIDs were reintroduced. Although the main objective considered sacroiliitis as present or absent, in reality, it is a continuum, and it is possible, therefore, that NSAID use decreases the appearance of active sacroiliitis but insufficiently to alter classification. Thus, the secondary objective was to determine the extent that NSAIDs affected the severity of active sacroiliitis using a semiquantitative scoring method. Furthermore, a subset of patients underwent quantitative MRI (qMRI) to provide an objective evaluation of any changes in inflammation with NSAIDs.

## PATIENTS AND METHODS

The DyNAMISM study (Do Non‐steroidal Anti‐inflammatory Drugs Mask Inflammation in Spondyloarthritis on MRI?) was a non‐randomized crossover study that recruited participants from NHS rheumatology clinics across the United Kingdom. Eligible participants were adults with established axSpA or suspected axSpA in whom a sacroiliac joint MRI scan was clinically indicated. In addition, they needed to be taking, or be about to commence, daily NSAIDs. To optimize the chances of observing active BME lesions, participants were required to have had a previous positive MRI result demonstrating active sacroiliitis and/or be male and HLA–B27 positive.^9^ Potential participants were excluded if they had received biologic therapy within the past six months, were currently receiving other anti‐inflammatory medication (eg, intramuscular or intravenous glucocorticoids), had any NSAID contraindications (eg, active peptic ulceration or severe hepatic or renal dysfunction) or contraindications to MRI (eg, having a metallic or conducting foreign body or severe claustrophobia), or were deemed potentially unable to understand the consent process.

Potential participants were invited to attend a baseline screening visit to confirm their eligibility. Clinical data were extracted from participants’ medical notes, including whether they fulfilled the modified New York classification criteria for ankylosing spondylitis^5^ and/or ASAS classification for axSpA^1^; presence or absence of spondyloarthritis features and extramusculoskeletal manifestations; and targeted medical history, including C‐reactive protein (CRP) level, year of symptom onset, and year first seen by a rheumatologist (a proxy for year of diagnosis). Participants were asked to complete questionnaires such as the Bath Ankylosing Spondylitis Disease Activity Index (BASDAI),^10^ which, along with patient global disease activity and CRP, allowed computation of the Ankylosing Spondylitis Disease Activity Score (ASDAS).^11^ Other measures included the Bath Ankylosing Spondylitis Patient Global Score,^12^ the Bath Ankylosing Spondylitis Functional Index,^13^ general health (Patient‐Reported Outcomes Measurement Information System Scale v1.2 Global Health),^14^, ^15^ disease‐specific quality of life measures (Ankylosing Spondylitis Quality of Life questionnaire),^16^ and the modified American College of Rheumatology preliminary diagnostic criteria for fibromyalgia.^17^

Participants were asked to discontinue all NSAID medication(s) for one week before undergoing a sacroiliac joint MRI scan (scan 1) using a standardized protocol including coronal‐oblique T1‐weighted and STIR sequences with 3‐mm slices.^18^ A one‐week washout period (equivalent to more than five half‐lives of the most commonly used NSAIDs) was thought to be sufficient based on drug pharmacokinetics (ie, unlike bisphosphonates, NSAIDs do not specifically target bone tissue). However, after approximately 20% of participants had been recruited, a protocol amendment was implemented to increase the washout period to two weeks because fewer participants than expected had a positive result at scan 1 and there was concern that the one‐week washout was not long enough.

All scans were anonymized and randomly allocated to two experienced readers (ANB, PMM, or HM‐O). Agreement between readers was examined using the kappa statistic, but in the event of any disagreement, a third reader adjudicated the result. Magnetic resonance images were accessed via a dedicated electronic platform created by the study coordinating center. Scans had a unique study identifier, and thus readers were blinded to participants’ clinical characteristics and scan date. On the first reading, active sacroiliitis was categorized as present (positive scan) or absent (negative scan) as per internationally recognized ASAS definitions, revised by Lambert et al.^19^ The amount of inflammatory signal required to define a positive scan result followed the specifications given by Rudwaleit et al^20^: if there was a single lesion suggesting active inflammation, it must be present on at least two consecutive MRI slices; if there was more than one lesion, then one slice may be sufficient.

Those with a positive baseline MRI finding (scan 1) were invited for a second MRI scan (scan 2) six weeks after restarting daily NSAIDs. By default, participants restarted whatever drug and dose they were on previously; changes were permitted but were not dictated by study protocol. For each participant, both scans were performed using the same MRI scanner and were again read independently by two readers, with adjudication if necessary. After the initial six‐week period (during which, by definition, all scans had to be scan 1), readers were also blinded to time point.

After study completion, all positive scans for scan 1 as well as all scans (positive or negative) for scan 2 were assessed for the degree of active sacroiliitis using the Leeds MRI scoring method, a semiquantitative approach that scores each joint quadrant using a combination of lesion size and intensity from 0 (no active sacroiliitis) to 3 (severe sacroiliitis), giving a theoretical range of 0 to 24, and where a BME lesion grading of ≥2 is considered clinically significant.^21^, ^22^ This secondary outcome was scored by one single reader, blind to scan chronology, and after first establishing that agreement between readers was excellent: the observed pairwise mean differences in scores were 0.15 (SD 2.33), −0.77 (SD 2.66), and −0.92 (SD 2.02) on the 0 to 24 scale (see Supplementary Figure 1).

Chemical shift‐encoded MRI (also known as Dixon MRI) was performed in a subset of patients to allow quantitative assessment of changes in fat content in the bone marrow, as fat content is known to reduce in the presence of BME.^23^, ^24^ Fat fraction (FF) maps were generated as described by Bainbridge et al^25^ and detailed in Supplementary Table 1. If specialist Dixon proton density FF packages such as mDixon Quant (Philips), Dixon FQ in the Liver Lab package (Siemens), or IDEAL IQ (GE) were available, these were used for the study. Alternatively, sites used a base‐level Dixon option such as mDixon (Philips), DIXON (Siemens), or LAVA FLEX (GE). The FF maps were analyzed using the semiautomated BEACH software tool^24^ by two readers (independent of the first two readers and adjudicator, with no access to their scores) who had been trained in the use of the tool. The BEACH tool was used to semiautomatically generate standardized regions of interest in the subchondral marrow of the sacroiliac joints; these regions were then analyzed to generate a set of histographic parameters for each patient at the 10th, 25th, 50th, 75th, and 90th percentiles (denoted FF_10_, FF_25_, FF_median_, FF_75_, and FF_90_, respectively). Over the pairs of scans, positive changes in FF parameters were taken to indicate improving inflammation and/or increasing fat metaplasia due to NSAIDs, whereas negative changes were taken to indicate worsening inflammation. As described previously,^24^ FF parameters targeting the lower end of the distribution (FF_10_ and FF_25_) may be more sensitive to edema or active inflammation, whereas those targeting the upper end of the distribution (FF_75_ and FF_90_) may be more sensitive to fat metaplasia, meaning that the contributions of these processes can be distinguished.

The primary analysis would determine the proportion of participants who changed from positive MRI results at scan 1 (not taking NSAIDs) to negative MRI results at scan 2 (taking NSAIDs). Jarrett et al reported MRI findings on 11 patients with r‐axSpA with inflammation in the sacroiliac joints.^6^ Of the nine with follow‐up MRI, three (33%) showed complete resolution with NSAIDs. The current study was powered on the more conservative estimate that 20% of participants would change from a positive MRI finding to a negative MRI finding after NSAIDs were reintroduced. Thus, 246 patients would be required to obtain a 95% confidence interval (CI) of ±5% around a prevalence estimate of 20%.

The proportion of participants who changed from positive to negative MRI findings was estimated along with an exact 95% CI. Factors associated with resolution of active sacroiliitis in scan 2 were examined using Poisson regression. Results are presented as risk ratios, with 95% CIs computed with robust SEs.

Leeds scoring was summarized using simple descriptive statistics, and agreement between MRI readers was examined using pairwise Bland–Altman difference plots.^26^ The change in qMRI parameters with NSAIDs was estimated using mixed‐effect linear regression modeling with maximum likelihood estimation; the fixed effects were the dependent (selected histographic parameter) and independent (treatment) variables, whereas the random effects were the patient and participant recruitment center. Z scores calculated from the regression were then used to determine one‐tailed *P* values (because of the specific directional hypothesis that NSAIDs would decrease inflammation). Interobserver agreement of qMRI parameters was evaluated using Bland–Altman limits of agreement analysis.

Data may be made available on reasonable request. The study was approved by West of Scotland Research Ethics Committee 3 (reference 17/WS/0041). All participants provided informed written consent.

## RESULTS

Between June 2017 and February 2020, 311 participants were recruited from 34 centers across England and Scotland. Participants had a median age of 42 years (interquartile range [IQR] 32–52), 62% were male, and the majority (87%) were of White racial origin. All had a clinical diagnosis of axSpA; 247 (79%) met the ASAS classification criteria for axSpA, of whom 107 (43%) had r‐axSpA. Median ASDAS and BASDAI scores were 2.8 (IQR 2.0–3.4) and 4.5 (IQR 2.8–6.2), respectively. Other baseline characteristics are shown in Table 1.

**Table 1:** Characteristics of study participants*

	Participants (N = 311)^**a**^	
	n (%)	Median (IQR)
Demographic characteristics		
Sex		
Female	117 (37.6)	–
Male	194 (62.4)	–
Age, y	–	42 (32–52)
Race and ethnicity		
White	271 (87.1)	–
Non‐White^b^	40 (4.2)	–
Clinical characteristics		
Symptom duration, y	–	9 (4–20)
Time since diagnosis, y	–	1 (0–7)
CRP ≥4 mg/L		
No	168 (54.0)	–
Yes	143 (46.0)	–
CRP,^c^ mg/L	–	6 (2.5–11)
Disease classification		
Radiographic axSpA	107 (34.4)	–
Non‐radiographic axSpA	140 (45.0)	–
Did not meet ASAS criteria	64 (20.6)	–
HLA–B27		
Positive	171 (55.0)	–
Negative	71 (22.8)	–
Unknown	69 (22.2)	–
Patient‐reported characteristics		
Disease activity		
ASDAS^c^	–	2.8 (2.0–3.4)
BASDAI	–	4.5 (2.8–6.2)
Function, BASFI	–	3.1 (1.5–5.6)
Global health		
BAS‐G	–	5 (3–6.5)
PROMIS physical^d^	–	15 (14–16)
PROMIS mental^d^	–	13 (11–16)
Quality of life, ASQoL	–	7 (3–12)
Fibromyalgia^e^		
Yes	68 (22.5)	–
No	234 (77.5)	–
Daily NSAIDs, before washout		
Yes	266 (85.5)	–
No	45 (14.5)	–

* ASAS, Assessment of SpondyloArthritis international Society; ASDAS, Ankylosing Spondylitis Disease Activity Score; ASQoL, Ankylosing Spondylitis Quality of Life questionnaire; axSpA, axial spondyloarthritis; BASDAI, Bath Ankylosing Spondylitis Disease Activity Index; BASFI, Bath Ankylosing Spondylitis Functional Index; BAS‐G, Bath Ankylosing Spondylitis Patient Global Score; CRP, C‐reactive protein; IQR, interquartile range; NSAID, nonsteroidal anti‐inflammatory drug; PROMIS, Patient‐Reported Outcomes Measurement Information System.

^a^ Precise denominator may vary due to missing data.

^b^ n = 13 Asian/Asian British (Indian, Pakistani, Bangladeshi); n = 3 Black British/Caribbean/Black African; n = 24 mixed or other.

^c^ Absolute CRP values were available only for 192 participants. This also influenced the number of participants for whom it was possible to compute an ASDAS value.

^d^ PROMIS global physical health and global mental health scales.

^e^ 2016 revisions to the 2010/2011 fibromyalgia diagnostic criteria.^17^

At baseline, most participants (83%) were taking naproxen, etoricoxib, or ibuprofen. A full list of NSAIDs is shown in Supplementary Table 2. Of the 311 participants, 286 (92%) completed the washout period and underwent scan 1. The reasons for failing to undergo scan 1 are shown in Table 2. The median washout duration was 17 (IQR 14–26) days. Fewer than half of participants reported a disease flare in the week before the scan (45%), although 69% experienced a worsening in disease activity and/or spinal pain. However, even in this group, the magnitude of worsening was modest; the median changes in the BASDAI and spinal pain scores were 0.8 (IQR 0.3–1.6) and 1 (IQR 0–2), respectively. The distribution of change in the BASDAI and spinal pain scores is shown in Supplementary Figure 2. Due to a high amount of missing data for CRP, it was only possible to calculate change in ASDAS for 148 participants. Mean change was 0.05 (95% CI −0.02 to 0.11), and only three participants reported a clinical important worsening over the washout period.^27^

**Table 2:** Reasons for dropout before scan 1*

	n
Failed washout attempt	
Could not tolerate washout period	2
Adverse event/required NSAID for other condition	4
Voluntary withdrawal from study^a^	1
Did not attend scan appointment	3
Other/unknown	15

* NSAID, nonsteroidal anti‐inflammatory drug.

^a^ Patient completed washout period and reported feeling very well. He was unwilling to recommence NSAIDs, as was required by study protocol.

Interreader agreement was good (pairwise comparisons ranged from 77% to 88%; κ = 0.53, 0.60, and 0.76), and fewer than 10% required third‐reader adjudication. In total, 146 participants (51%) had a positive scan result for active sacroiliitis at scan 1, and a follow‐up scan (while taking NSAIDs) was obtained from 124 (85% of those eligible). The median between‐scan interval was 42 (IQR 42–48) days. Twenty‐five participants (20.2%) had a negative scan result for active sacroiliitis (95% CI 13.5%–28.3%).

There was no evidence of a sex difference in the risk of scoring negative for sacroiliitis while taking NSAIDs (risk ratio [male vs female] 1.25; 95% CI 0.59–2.68). Indeed, there were no significant associations between any demographic, clinical, or patient‐reported characteristics and the risk of sacroiliitis after recommencing NSAIDs (see Table 3).

**Table 3:** Factors associated with a positive or negative scan result while taking NSAIDs*

	Scan 2, sacroiliitis, n (%)		RR^b^ (95% CI)
	Positive (n = 99)^a^	Negative (n = 25)^a^	
Demographic characteristics			
Sex			
Female	38 (82.6)	8 (17.4)	–
Male	61 (78.2)	17 (21.8)	1.25 (0.59–2.68)
Age			
≤39 y	52 (83.9)	10 (16.1)	–
≥40 y	47 (75.8)	15 (24.2)	1.50 (0.73–3.09)
Race and ethnicity			
White	85 (78.7)	23 (21.3)	–
Non‐White	14 (87.5)	2 (12.5)	0.57 (0.15–2.27)
Clinical characteristics			
Symptom duration			
≤8 y	52 (82.5)	11 (17.5)	–
≥9 y	47 (78.3)	13 (21.7)	1.24 (0.60–2.56)
Time since diagnosis			
≤1 y	50 (80.6)	12 (19.4)	–
≥2 y	49 (80.3)	12 (19.7)	1.02 (0.49–2.09)
CRP ≥4 mg/L			
No	48 (78.7)	13 (21.3)	–
Yes	51 (81.0)	12 (19.0)	0.89 (0.44–1.81)
ASAS axSpA criteria			
No	13 (65.0)	7 (35.0)	–
Yes	86 (82.7)	18 (17.3)	0.49 (0.24–1.03)
Radiographic axSpA			
No	71 (82.6)	15 (17.4)	–
Yes	28 (73.7)	10 (26.3)	1.51 (0.74–3.06)
HLA–B27			
Positive	60 (83.3)	12 (16.7)	–
Negative	23 (82.1)	5 (17.9)	1.07 (0.41–2.77)
Unknown	16 (66.7)	8 (33.3)	2.00 (0.93–4.32)
Patient‐reported characteristics			
Disease activity			
ASDAS			
≤3.99	34 (79.1)	9 (20.9)	–
≥4.00	36 (83.7)	7 (16.3)	0.78 (0.32–1.91)
BASDAI			
≤4.4	53 (85.5)	9 (14.5)	–
≥4.5	45 (73.8)	16 (26.2)	1.81 (0.86–3.78)
Function, BASFI			
≤2.7	52 (83.9)	10 (16.1)	–
≥2.8	46 (75.4)	15 (24.6)	1.52 (0.74–3.13)
Global health			
BAS‐G			
≤4.5	54 (84.4)	10 (15.6)	–
≥5.0	44 (74.6)	15 (25.4)	1.63 (0.79–3.34)
PROMIS physical^c^			
≤15	63 (77.8)	18 (22.2)	–
≥16	35 (83.3)	7 (16.7)	0.75 (0.34–1.66)
PROMIS mental^c^			
≤13	50 (79.4)	13 (20.6)	–
≥14	48 (80.0)	12 (20.0)	0.97 (0.48–1.96)
Quality of life, ASQoL			
≤6	51 (79.7)	13 (20.3)	–
≥7	47 (79.7)	12 (20.3)	1.00 (0.50–2.02)
Fibromyalgia^d^			
No	81 (80.2)	20 (19.8)	–
Yes	17 (77.3)	5 (22.7)	1.15 (0.48–2.73)
Duration of NSAID washout			
NSAID washout			
≤14 days^e^	10 (25.0)	30 (75.0)	–
15–21 days	8 (17.8)	37 (82.2)	0.71 (0.31–1.63)
≥22 days	7 (17.9)	32 (82.1)	0.72 (0.30–1.70)

*Note*: Asian/Asian British (Indian, Pakistani, Bangladeshi); Black British/Caribbean/Black African; or Other.

* ASAS, Assessment of SpondyloArthritis international Society; ASDAS, Ankylosing Spondylitis Disease Activity Score; ASQoL, Ankylosing Spondylitis Quality of Life questionnaire; axSpA, axial spondyloarthritis; BASDAI, Bath Ankylosing Spondylitis Disease Activity Index; BASFI, Bath Ankylosing Spondylitis Functional Index; BAS‐G, Bath Ankylosing Spondylitis Patient Global Score; CI, confidence interval; CRP, C‐reactive protein; NSAID, nonsteroidal anti‐inflammatory drug; PROMIS, Patient‐Reported Outcomes Measurement Information System; RR, risk ratio.

^a^ Precise denominator may vary due to missing data.

^b^ RR and 95% CI given per category. The reference category is always listed first. Continuous variables have been categorized and split above or below the median value due to violations of linearity assumptions.

^c^ PROMIS global physical health and global mental health scales.

^d^ 2016 revisions to the 2010/2011 fibromyalgia diagnostic criteria.^17^

^e^ Includes 5 participants who had a washout duration of ≤7 days and 35 who had a washout duration of 8–14 days.

### Semiquantitative scoring (Leeds MRI scoring method)

The median total Leeds scores were 4 (IQR 2–8) and 3 (IQR 2–7) for scan 1 and scan 2, respectively. While taking NSAIDs, more than two‐fifths of participants experienced a reduction in the Leeds score (44%; 95% CI 35%–54%), with a median change of −2 (IQR −3 to −1).

### qMRI analysis

**Figure 1:**
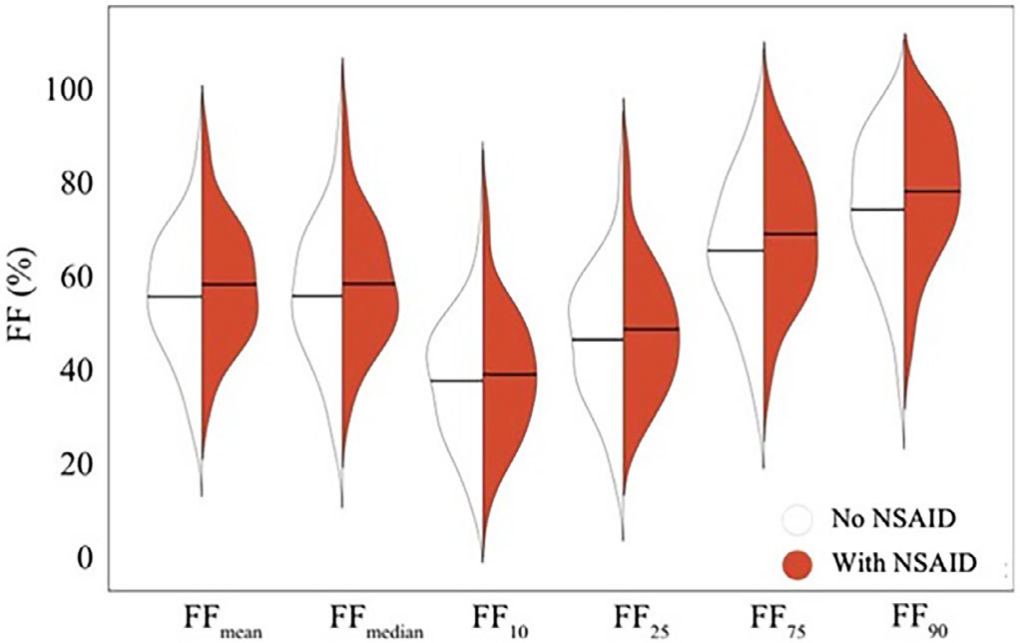
Violin plots demonstrating changes in quantitative magnetic resonance imaging parameters. FF‐based histographic parameters are shown from before and after treatment. Note that the variability in the baseline FF values is effectively dealt with in the mixed‐effect linear regression because each patient acts as their own control. FF, fat fraction; NSAID, nonsteroidal anti‐inflammatory drug.

One hundred four patients underwent pre‐NSAID Dixon scans for FF measurement, of whom 39 had repeat scans. Changes in FF histographic parameters with NSAIDs are summarized in Table 4 and Figure 1. FF measurements were higher on scans acquired while participants were taking NSAIDs compared to the scans acquired while participants were not taking NSAIDs, with significant increases observed for FF_75_ and FF_90_. The observed increases in the values of FF_10_, FF_25_, and FF_median_ were numerically smaller than those in FF_75_ and FF_90_ and did not reach statistical significance. Bland–Altman limits of agreement analysis showed good interobserver agreement generally, for all histographic parameters (example plots are shown in Supplementary Figure 3).

**Table 4:** Change in qMRI parameters while taking NSAIDs*

FF^a^	Change with NSAID, β coefficient^b^	95% CI^b^	Z score^b^	*P* value
FF_mean_	0.550	−0.215 to 1.315	1.51	0.080
FF_median_	0.568	−0.233 to 1.369	1.39	0.082
FF_10_	0.086	−0.928 to 1.010	0.17	0.434
FF_25_	0.495	−0.370 to 1.360	1.12	0.131
FF_75_	0.809	−0.065 to 1.683	1.81	0.035^c^
FF_90_	0.916	0.056 to 1.776	2.09	0.019^c^

* Comparison of histographic parameters obtained between pre‐ and posttreatment patients. CI, confidence interval; FF, fat fraction; NSAID, nonsteroidal anti‐inflammatory drug; qMRI, quantitative magnetic resonance imaging.

^a^ Subscript numbers denote the 10th, 25th, 75th, and 90th percentiles of FF in the defined regions of interest.

^b^ Coefficient, 95% CIs, and Z values were derived from the mixed‐effect linear regression.

^c^ *P* values were calculated separately and refer to a one‐tailed z test.

## DISCUSSION

This study demonstrates that among patients with axSpA with evidence of active sacroiliitis after an NSAID washout, one in five exhibit resolution of sacroiliitis six weeks after NSAIDs have been reintroduced. This provides evidence of the effect of NSAIDs on active sacroiliitis and shows that NSAID use before MRI may have an important impact on clinical diagnosis. Further, we show that although patients may experience a deterioration in disease activity and spinal pain during NSAID washout, almost all patients who attempted NSAID washout were able to tolerate it. Thus, stopping NSAIDs for one to two weeks before MRI scanning in clinical practice should be considered.

There are a number of methodologic issues to discuss. Firstly, it would have been possible to answer the research question with a randomized crossover trial. However, this was discounted for reasons of inefficiency and concerns over participant well‐being. For example, some participants would have a negative scan result at both time points and would not contribute any data to the main research question despite having undergone the entire study protocol, including two MRI scans and an NSAID washout period. The real challenge of interpretation therefore is whether change in sacroiliitis after NSAID reintroduction might be due to the natural course of MRI inflammation. It would have been interesting to rescan some participants six weeks after a positive scan result for sacroiliitis but without restarting NSAIDs. This was considered and would have provided some data on the natural history of sacroiliitis but was discounted; it would have been necessary to ask participants to remain off their usual medication for four times longer than was necessary to answer the main research question. This was felt not to be justifiable from an ethical perspective.

The pertinent issue, however, is whether the change in sacroiliitis at scan 2 could be explained by natural variation in sacroiliitis. The results might be explained by regression to the mean if study participants were all recruited at a time of high disease activity (assuming corresponding active inflammatory lesions). However, there was no significant difference in the proportion of participants who had a negative scan result at scan 2 between those who did versus did not report a flare at the time of scan 1. Current evidence on the natural variation of sacroiliitis over the short term remains scarce. A recent study showed that short‐lived fluctuations within a few days in MRI‐determined BME were more common with longer‐acting TNF inhibitors and corresponded with a subjective loss of clinical response before the next scheduled dose.^28^ However, in a previous study, 29 participants with normal sacroiliac joint radiographs who fulfilled the ASAS criteria for inflammatory back pain underwent four MRI scans over a 12‐week period. Results showed that of the 10 who had a positive result for inflammation in the spine and/or sacroiliac joints, all these participants had subsequent positive results at 4, 8, and 12 weeks.^29^ Nearly half the patients continued their regular NSAID intake during the study, and none were receiving biologic therapy. Thus, although it is possible that the resolution of inflammatory lesions in the DyNAMISM study (over a six‐week period) occurred spontaneously, this earlier study suggests that rapid fluctuation from a positive to a negative result for sacroiliitis is uncommon. Hence, we believe that the observed changes in the current study can reasonably be attributed to NSAIDs.

It is important to highlight that the DyNAMISM study population is predominantly male. This is due, at least in part, to the fact that in the early part of recruitment, we prioritized men who were HLA–B27 positive to increase study efficiency; this is a subgroup known to have higher likelihood of a positive sacroiliac joint MRI finding.^9^ Nevertheless, this criterion was broadened during the study to include anyone (male or female) with a prior positive MRI finding. In terms of external validity, there is no reason to believe that if NSAIDs affect appearance of MRI in men why this would not also be the same in women, an assumption that was borne out by the results.

Unfortunately, 22% of patients were missing data on HLA–B27 status, although this was expected because HLA–B27 was not a protocol‐mandated test, and other studies have had similar findings. For example, in the British Society for Rheumatology Biologics Register for Ankylosing Spondylitis, a large registry that recruited from around 80 hospitals across Great Britain, >40% of participants were missing information on HLA–B27.^30^ Pertinent to the current study, although knowledge of HLA–B27 status might influence diagnosis and can definitely influence disease classification, it will not necessarily influence the decision of whether a patient is referred for imaging. Further, HLA–B27 status was not a determinant of whether a patient changed status (from positive to negative on MRI).

At recruitment, all participants were taking (or were to commence) daily NSAIDs, and after scan 1, all restarted (or commenced) daily NSAIDs. Scan 2 was undertaken six weeks subsequently. ASAS/EULAR recommendations for the management of axSpA recommend that before escalating to biologic or targeted synthetic disease‐modifying antirheumatic drugs, nonpharmacological treatments and at least two NSAIDs should be tried over a four‐week period.^4^ We would argue that if four weeks is sufficient to determine effectiveness of NSAIDs in a management context, six weeks should be sufficient to observe any effect on active sacroiliitis should such an effect exist. It is possible that the full effect of NSAIDs on active sacroiliitis may take longer, but if this is the case, then the actual effect observed in the current study would be an underestimate.

From a clinical perspective, a potential concern is whether patients can tolerate an NSAID washout period in the first place. The majority of patients in the DyNAMISM study reported an increase in disease activity and/or spinal pain during the washout period, and 45% of participants reported a flare in the week before scan 1. However, increases in disease activity and spinal pain were relatively small; even among those with a deterioration in BASDAI and/or spinal pain scores, the median increase was ≤1 point. Reassuringly, almost all participants (>90%) who attempted the NSAID washout were able to achieve it, and in the subgroup with ASDAS measurements both before and after washout, only a small minority (2%) reported a clinically important worsening.

The DyNAMISM study protocol did not stipulate the specific NSAID treatment. Instead, participants took the brand and dose as prescribed by their rheumatologist, reflecting real‐world clinical practice. The half‐life of NSAIDs vary, from <1 hour^31^ to around 48 hours,^32^ so it is possible that if a washout period is too short, the drugs might still be exerting an effect, increasing the chance of a negative scan result. In contrast, if the washout period is too long, although increasing the chances of patients having a positive scanning result (if indeed the results are positive), there may be implications for patient comfort and safety. In the current study, very few participants (<5%) had an NSAID washout period of less than one week, and it was not meaningful to examine this group separately. Instead, the study population was divided into washout periods of ≤14 days, 15 to 21 days, and ≥22 days. There was no evidence that a longer washout duration was associated with response (a negative result at scan 2) after NSAIDs were reintroduced. The current study provides no evidence of any benefit of extending the washout beyond 14 days.

We were unable to identify any clinical characteristics that would indicate, in advance, who might be likely to change status (from positive for sacroiliitis to negative). Some small differences were evident, although there were no consistent or significant differences between groups, but the study was not specifically powered to detect such differences. It was interesting that compared to those who did not, participants who met ASAS axSpA classification criteria were half as likely to have a negative scan result in the presence of NSAIDs. Despite this, still around one in five participants changed status after NSAIDs were reintroduced. The high proportion of missing data for HLA–B27 status has already been discussed. However, it is interesting to note that among those for whom data were available, there was no difference in the proportion who scanned positive at scan 2 between those who were B27 positive versus negative (83.3% vs 82.1%).

In the current study, for the primary outcome, all scans were read by two readers (with adjudication by a third, if required). All were rheumatologists with a specialist interest in axSpA and experience in applying the ASAS criteria for active inflammatory lesions (positive MRI finding) of the sacroiliac joint.^20^ Interreader agreement was good, with only 12% of all scans requiring adjudication. Reliability in reading the Leeds MRI scoring method was also examined. Here, each quadrant of each sacroiliac joint is scored from 0 to 3, giving a total score of 0 to 24. Although it is harder to gain agreement on a semiquantitative scale, interreader reliability was excellent. The Leeds method was chosen because of its feasibility, reader familiarity, and the fact that it incorporates a subjective assessment of extent and intensity of the BME lesion as a proxy of lesion severity. Indeed, results from the Leeds scoring method showed that 44% of participants exhibited a reduction in BME severity in the presence of NSAIDs, more than twice the proportion who changed status from ASAS positive to ASAS negative. This suggests that NSAIDs have an effect on decreasing the severity of inflammation, although this may not always be sufficient to fully suppress it.

To explore this effect further, a subset of participants underwent qMRI. This novel feature of the study provides quantitative support for the hypothesis that NSAIDs reduce inflammation on MRI. We observed larger increases in the parameters targeting the upper “fatty” end of the distribution than in the parameters targeting the lower “inflamed” end of the distribution, suggesting an emergence or “uncovering” of fat metaplasia in the bone marrow of participants not taking NSAIDs. Further qMRI studies may help to define the response phenotype more precisely. In general, qMRI analyses may provide useful secondary end points in clinical studies, as the subjectivity inherent to conventional MRI reading is minimized. A further benefit is that with emerging techniques, MRI changes due to BME can potentially be disentangled from those due to structural damage, which can otherwise confound interpretation.^33^ The use of these qMRI techniques and/or artificial intelligence–based methods could allow a more detailed analysis clarifying the role of NSAIDs in modulating BME and also its impact on disease progression in axSpA.^34^

In conclusion, these findings provide important insights on the impact of NSAIDs on the appearance of MRI‐determined BME lesions representative of active sacroiliitis in axSpA. Significant improvements in the severity of the lesions were observed in >40% of participants, with full resolution seen in 20%. Some patients will be unwilling to attempt an NSAID washout, fearful of the increase in pain and disease activity. However, this study also provides reassuring evidence that among patients willing to attempt a washout, almost all can successfully achieve it. These data have important implications for axSpA diagnosis and classification and, in clinical practice, support consideration of a 14‐day NSAID washout before MRI.

## AUTHOR CONTRIBUTIONS

All authors contributed to at least one of the following manuscript preparation roles: conceptualization AND/OR methodology, software, investigation, formal analysis, data curation, visualization, and validation AND drafting or reviewing/editing the final draft (A CRediT statement detailing author contributions is shown in Supplementary Table 3a/3b). As corresponding author, Dr Jones confirms that all authors have provided the final approval of the version to be published and takes responsibility for the affirmations regarding article submission (eg, not under consideration by another journal), the integrity of the data presented, and the statements regarding compliance with institutional review board/Declaration of Helsinki requirements.

## Supporting information


**Disclosure Form**:

**Data S1** Supporting Information
