# Supplementary material for: Effect of Nonsteroidal Anti‐Inflammatory Drugs on Sacroiliac Joint Inflammation, as Seen on Magnetic Resonance Imaging, in Axial Spondyloarthritis
Source: Arthritis Care Res (Hoboken). 2025 Sep 10;77(12):1443–52. doi: 10.1002/acr.25581 (PMC12750118; doi:10.1002/acr.25581)
Supplement: Supplementary file 2 — Data S1 Supporting Information [file ACR-77-1443-s002.pdf]

Supplementary Figure 1

Bland-Altman plots illustrating agreement between MRI readers (Leeds MRI Scoring method)

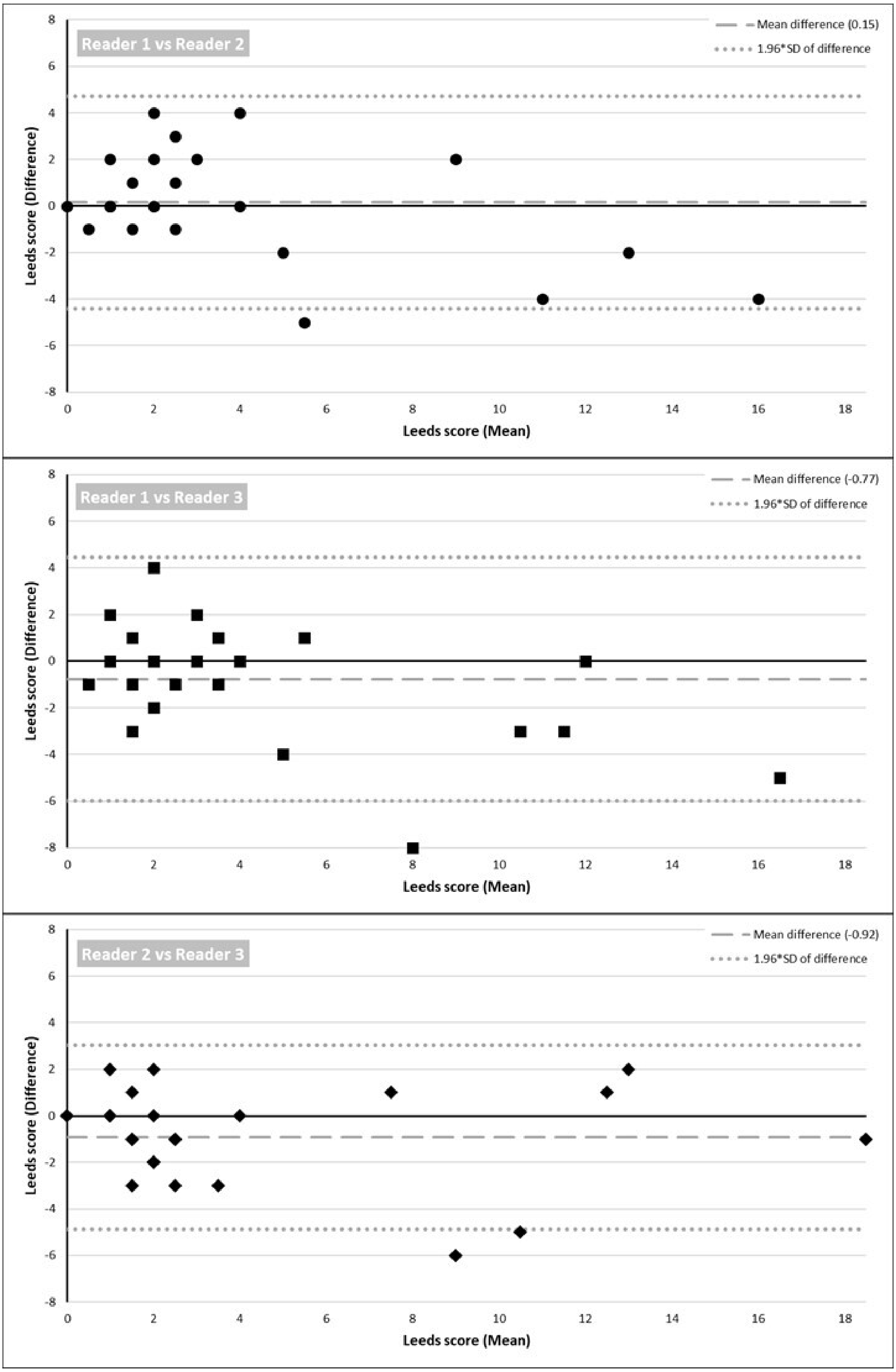

Supplementary Figure 2

Change in BASDAI (a), and spinal pain (b), during NSAID washout

(a)

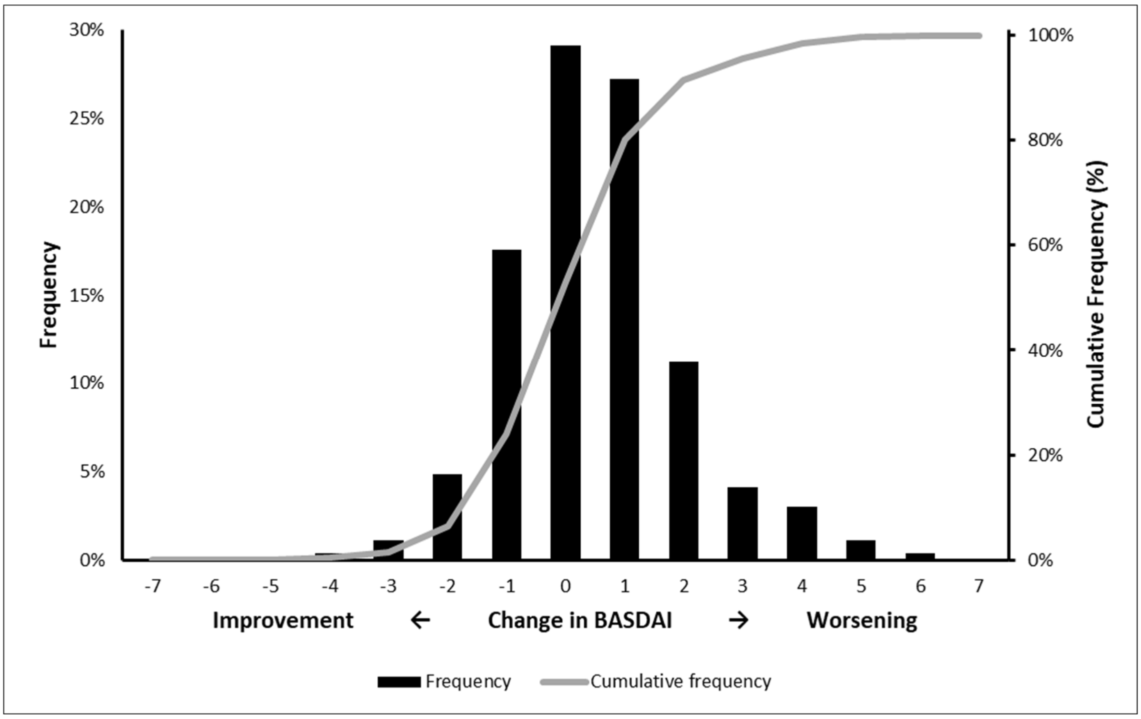

(b)

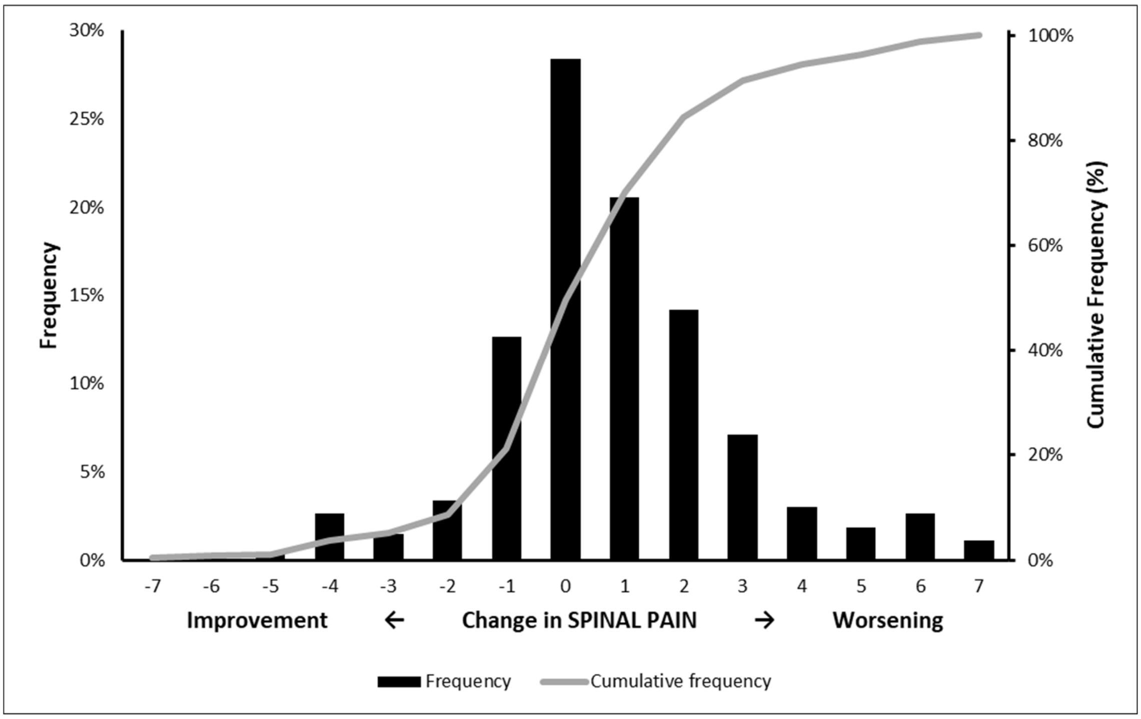

Supplementary Figure 3

Bland-Altman limits of agreement analysis for FF_{90} (a), and FF_{10} (b)

(a)

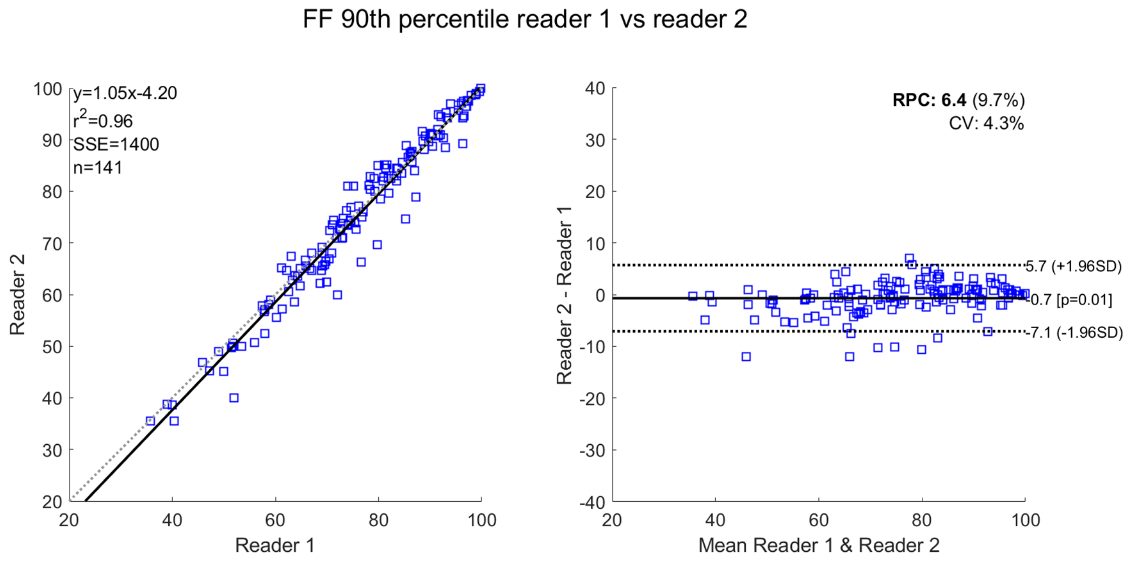

(b)

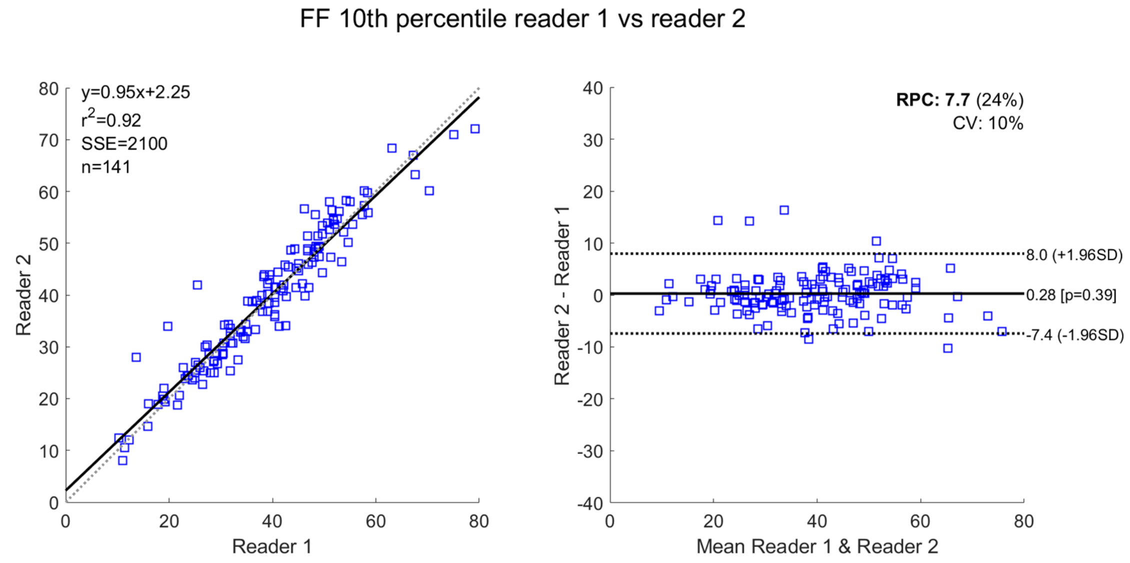

Supplementary Table 1

The range of parameters used for Dixon acquisitions

Parameter	Specialist package range	Base-level Dixon range
**Field Strength**	1.5 T	1.5 T
**Orientation**	Paracoronal	Paracoronal
**Acquisition**	3D Spoiled Gradient Echo	3D Spoiled Gradient Echo
**Acquisition Time**	20-40s	20-40s
**3d Slab Dimensions**	In-plane: 490 * 360 mm<br>Through plane: 100 mm<br>These dimensions can be adjusted if necessary to reduce imaging time	In-plane: 490 * 360 mm<br>Through plane: 100 mm<br>These dimensions can be adjusted if necessary to reduce imaging time
**Acquisition Matrix**	In Plane: 256 * 192	In Plane: 256 * 192
**Slice Thickness**	4 mm	4 mm
**TR**	Shortest (5-10ms)	Shortest (5-10ms)
**Number of echoes**	6	2 or 3
**TE of first echo**	Shortest (1-2ms)	n/a
**Echo Spacing**	Shortest (1-2ms)	n/a
**Echo times**	n/a	In phase / out phase or minimum as appropriate for Dixon method
**Flip Angle**	3 degrees	3 degrees
**Number of averages**	1	1
**Parallel imaging**	Up to speed-up factor 2	Up to speed-up factor 2
**Reconstructed images**	Fat only image<br>Water only image<br>T2* or R2* map (if possible)<br>Fat Fraction map (if possible)	Fat only image<br>Water only image<br>Fat Fraction image (if possible)

Supplementary Table 2

Non-Steroidal Anti-Inflammatory Drugs reported at recruitment (as self-reported by study participants)

NSAID	Prescribed	Over-the-counter
Naproxen	86	6
Etoricoxib	74	2
Ibuprofen	42	51
Celecoxib	20	
Etodolac	11	
Diclofenac	8	1
Meloxicam	4	
Nabumetone	3	
Diclofenac / misoprostol	1	
Ibuprofen / codeine	1	1
Indomethacin	1	1
Piroxicam	1	
>1 Prescribed NSAID	4[1]	
Other		1[2]

1 Were listed in the medical notes as: Etoricoxib + Celecoxib | Etoricoxib + Naproxen | Ibuprofen + Diclofenac | Naproxen + Ibuprofen
2 Was reported by the participant as: Ibuprofen / Naproxen / Indomethacin.

Supplementary Table 3a

CRediT statement, author contributions (Contributor Roles Taxonomy; https://credit.niso.org)

	Conceptualisation	Methodology	Software	Validation	Formal analysis	Investigation	Resources	Data curation	Writing (Original draft)	Writing (Review and editing)	Visualisation	Supervision	Project administration	Funding acquisition
Authors														
Gareth T Jones	✓	✓			✓	✓			✓	✓	✓	✓	✓	✓
Alex Bennett	✓	✓				✓				✓		✓	✓	✓
Raj Sengupta	✓	✓				✓				✓		✓	✓	✓
Pedro Machado						✓				✓				
Helena Marzo-Ortega						✓				✓				
Lorna Aucott		✓								✓		✓	✓	
Margaret Hall-Craggs	✓									✓		✓		✓
Timothy Bray	✓		✓		✓				✓	✓				✓
Alan Bainbridge			✓					✓		✓		✓		✓
Ruaridh Gollifer					✓			✓	✓	✓				
Gary J Macfarlane	✓	✓								✓		✓	✓	✓
Non-author contributors														
Karen Forrest Keenan						✓							✓	
Laura Moir						✓							✓	
Dolapo Ayansina						✓								

Supplementary Table 3b

CRediT statement, contribution descriptors

Term	Description
Conceptualisation	Ideas; formulation or evolution of overarching research goals and aims
Methodology	Development or design of methodology; creation of models
Software	Programming, software development; designing computer programs; implementation of the computer code and supporting algorithms; testing of existing code components
Validation	Verification, whether as a part of the activity or separate, of the overall replication/reproducibility of results/experiments and other research outputs
Formal analysis	Application of statistical, mathematical, computational, or other formal techniques to analyse or synthesize study data
Investigation	Conducting a research and investigation process, specifically performing the experiments, or data/evidence collection
Resources	Provision of study materials, reagents, materials, patients, laboratory samples, animals, instrumentation, computing resources, or other analysis tools
Data curation	Management activities to annotate (produce metadata), scrub data and maintain research data (including software code, where it is necessary for interpreting the data itself) for initial use and later reuse
Writing (Original draft)	Preparation, creation, and/or presentation of the published work, specifically writing the initial draft (including substantive translation)
Writing (Review and editing)	Preparation, creation, and/or presentation of the published work by those from the original research group, specifically critical review, commentary, or revision – including pre- or post-publication stages
Visualisation	Preparation, creation, and/or presentation of the published work, specifically visualization/ data presentation
Supervision	Oversight and leadership responsibility for the research activity planning and execution, including mentorship external to the core team
Project administration	Management and coordination responsibility for the research activity planning and execution
Funding acquisition	Acquisition of the financial support for the project leading to this publication
